# Acute and Long-Term Hemodynamic Effects of MitraClip Implantation on a Preexisting Secondary Right Heart Failure

**DOI:** 10.1155/2018/6817832

**Published:** 2018-03-14

**Authors:** M. Hünlich, E. Lubos, B. E. Beuthner, M. Puls, A. Bleckmann, T. Beißbarth, T. Tichelbäcker, V. Rudolph, S. Baldus, U. Schäfer, H. Treede, R. S. Von Bardeleben, S. Blankenberg, W. Schillinger

**Affiliations:** ^1^Herzzentrum, Abt. Kardiologie und Pneumologie, Universitätsklinikum Göttingen, Göttingen, Germany; ^2^Klinik und Poliklinik für Allgemeine und Interventionelle Kardiologie, Universitäres Herzzentrum Hamburg GmbH, Hamburg, Germany; ^3^Institut für Medizinische Statistik, Universitätsmedizin Göttingen, Göttingen, Germany; ^4^Klinik für Kardiologie, Angiologie, Pneumologie und Internistische Intensivmedizin, Herzzentrum der Universität zu Köln, Köln, Germany; ^5^Universitätsklinik und Poliklinik für Herzchirurgie, Martin-Luther-Universität, Halle-Wittenberg, Halle, Germany; ^6^II. Medizinische Klinik und Poliklinik, Universitätsmedizin der Johannes-Gutenberg-Universität, Mainz, Germany

## Abstract

Positive results of MitraClip in terms of improvement in clinical and left ventricular parameters have been described in detail. However, long-term effects on secondary pulmonary hypertension were not investigated in a larger patient cohort to date. 70 patients with severe mitral regurgitation, additional pulmonary hypertension, and right heart failure as a result of left heart disease were treated in the heart centers Hamburg and Göttingen. Immediately after successful MitraClip implantation, a reduction of the RVOT diameter from 3.52 cm to 3.44 cm was observed reaching a statistically significant value of 3.39 cm after 12 months. In contrast, there was a significant reduction in the velocity of the tricuspid regurgitation (TR) from 4.17 m/s to 3.11 m/s, the gradient of the TR from 48.5 mmHg to 39.3 mmHg, and the systolic pulmonary artery pressure (PAPsyst) from 58.6 mmHg to 50.0 mmHg. This decline continued in the following months (V_max_ TR 3.09 m/s, peak TR 38.6 mmHg, and PAPsyst 47.4 mmHg). The tricuspid annular plane systolic excursion (TAPSE) increased from 16.5 mm to 18.9 mm after 12 months. MitraClip implantation improves pulmonary artery pressure, tricuspid regurgitation, and TAPSE after 12 months. At the same time, there is a decrease in the RVOT diameter without significant changes in other right ventricular and right atrial dimensions.

## 1. Introduction

Mitral regurgitation is the second most common cause of heart valve failure in Europe [1] and leads to a continuous backflow of blood into the pulmonary vessels.

While an acute pressure increase in the pulmonary veins leads to pulmonary edema, chronic congestion often ends in reactive pulmonary vasoconstriction with consecutive secondary pulmonary hypertension. Alongside the degree of mitral regurgitation, other factors, such as severe impaired left ventricular ejection fraction, may also have an impact on secondary pulmonary hypertension [2–4].

Previous published data demonstrate an important prognostic effect on perioperative course of right ventricular function and hemodynamics in patients with impaired left ventricular ejection fraction and pulmonary venous hypertension [5, 6]. Echocardiography provides a noninvasive standard method for determination of right ventricular functional parameters. Due to its complex geometry, the ejection fraction of the right ventricle cannot be easily quantified by echo. In daily routine, the end-diastolic and end-systolic volumes in the apical four-chamber view are determined and from this, the right ventricular ejection fraction is usually calculated, though it can easily lead to misinterpretation of both ejection fraction and RV volumes [7]. In contrast, a very good correlation with the right ventricular ejection fraction is possible from the determination of the movement of the tricuspid valve ring. Unlike the left ventricle, the right ventricle consists primarily of longitudinal muscle fibers, leading to a marked basoapical movement of the tricuspid valve ring (TAPSE) during systole. Movement restrictions of less than 17 mm are regarded as pathological [8]. In addition, the systolic and mean pulmonary artery pressure can be determined, which correlate very well with invasively measured values [9].

Patients with chronic heart failure due to ischemic or dilated cardiomyopathy often undergo surgical revascularization or cardiac valve surgery to improve the left ventricular function. In large surgical cohorts, an influence of the secondary right heart failure solely by correction of mitral valve insufficiency was often associated with inconstant outcomes and increased mortality and morbidity [10–12].

Positive results of a MitraClip implantation in terms of an improvement in clinical and left ventricular parameters have been described in detail in the current literature [13, 14]. Pulmonary hypertension and/or right heart failure are an important and frequently encountered clinical scenario in patients with severe mitral regurgitation [15, 16]. However, long-term effects of a minimally invasive Mitral Valve Repair using MitraClip on secondary pulmonary hypertension were not investigated in a larger patient cohort to date.

## 2. Methods

Patients with severe mitral regurgitation have been treated in the heart centers of Hamburg and Göttingen since 2009, using the MitraClip procedure. Anonymized clinical and epidemiological data were scientifically evaluated after written informed consent by the patient. The current study was done in accordance with national and international guidelines and the Declaration of Helsinki after approval by the local ethical committees.

### 2.1. Inclusion and Exclusion Criteria

Between 2009 and 2015, 70 patients with severe mitral regurgitation were treated in both cardiac centers, using MitraClip where additional pulmonary hypertension was present due to left heart disease. In addition, clinical signs of right heart failure, for example, edema or aszites, had to exist. Patients with pulmonary hypertension or right heart failure from other causes such as chronic thromboembolism (CTEPH) or unclear or multifactorial mechanisms were excluded from the current analysis.

### 2.2. Specific Inclusion/Exclusion Criteria

To estimate the pulmonary artery pressure, the presence of a measureable tricuspid regurgitation was necessary. Additionally, evaluable echocardiographic data for each patient had to be available, prior the MitraClip implantation, before hospital discharge after successful implantation, and again after twelve months.

### 2.3. Echocardiography

All transthoracic echocardiography readings were made during clinical routine and digitally stored, so that subsequent measurements and evaluations were possible. A retrospective echocardiographic analysis took place in accordance with the recommendations of the American and European scientific societies [17, 18]. Particular attention was being paid to the right ventricular parameters by two independent investigators to whom patient names and time point of examination (before the MitraClip implantation, before discharge after successful MitraClip implantation, or twelve months after the implantation) were not revealed. Given the variation of particular RV und TV measurements on echocardiography, an inter- and intraobserver variability analysis was performed which revealed no significant difference.

RV dimensions and tricuspid valve parameters were quantified from the apical four-chamber view. LVOT diameters were measured in the parasternal outflow view and inferior vena cava diameters in the subcostal view.

The estimation of systolic pulmonary artery pressure (PAPsyst) was carried out by a continuous-wave Doppler determination of the maximum velocity of the tricuspid regurgitation (*V*_max_) and this value was used in the modified Bernoulli equation (PAPsyst = 4  *∗*  *V*_max_ + medium right atrial pressure). For the right atrial pressure (RAP), a value of 5 mmHg at complete collapse of the inferior vena cava was applied as well as a value of 10 mmHg at a partial collapse and a value of 15 mmHg in the absence of collapse [19]. Mean pulmonary artery pressure (MPAP) was calculated by tricuspid regurgitation jet velocity-time integral (TR VTI) and RAP using the following formula: mPAP = mean  Δ*P* + RAP [20]. In accordance with current ESC and AHA/ACC guidelines, a pulmonary hypertension was assumed with a mean pulmonary pressure value greater than 25 mmHg at rest [17, 18, 21]. In addition, PAPsyst values > 50 mmHg were used as cut-off since Barbieri et al. showed an increased mortality of patients with severe mitral regurgitation [11].

### 2.4. Statistical Analysis

Statistical analysis was performed with the Statistical Computing Software R (version 2.15.1; https://www.r-project.org). Differences in echocardiographic measurements were calculated using Wilcoxon paired signed-rank test. A value of *P* < 0.05 was considered statistically significant.

## 3. Results

### 3.1. Baseline Characteristics

The demographic and clinical characteristics of the patients at the inclusion date are summarized in Table 1. The average age was 72.5 years, with a proportion of 66% male patients. The secondary mitral regurgitation predominated with 71%. All patients had symptomatic heart failure predominantly in NYHA stages III and IV (94%). At the same time, multiple comorbidities existed in the patients studied, in particular impaired renal function (66%) and atrial fibrillation (64%). However, coronary heart disease (36%) and diabetes mellitus (27%) were often observed, too. The comorbidities mentioned above contributed to the very high mean logistic Euro score of 30% and STS score of 10%. The average distance walked in the six-minute walk test was determined to be 213 meters.

### 3.2. Diameter and Area of the RA, RV, and the Vena Cava Inferior

The exact echocardiographic readings are summarized in the first part of Table 2.

**Figure 1:**
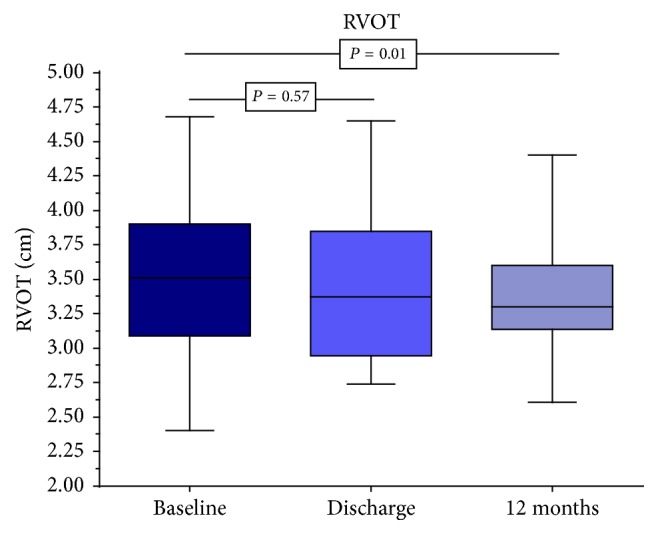
Significant decrease of right ventricular outflow tract (RVOT) diameter from 3.52 cm to 3.39 cm after twelve months.

Both the basal and the middle and longitudinal diameters of the RV and the diameter of the RA compared to baseline did not change significantly in the follow-up period. The area measurements of the RA and RV and the width of the vena cava inferior also remained unchanged over 12 months. Immediately after successful MitraClip implantation, a reduction of the RVOT diameter of 3.52 cm to 3.44 cm was observed in contrast to the above parameters. However, this decrease was not yet significant at the time of discharge. In the further course of time, the diameter decreased further, reaching a statistically significant value of 3.39 cm after 12 months (Figure 1).

### 3.3. Echocardiographic Changes to the TV

The exact echocardiographic measurements are summarized in the second part of Table 2.

**Figure 2:**
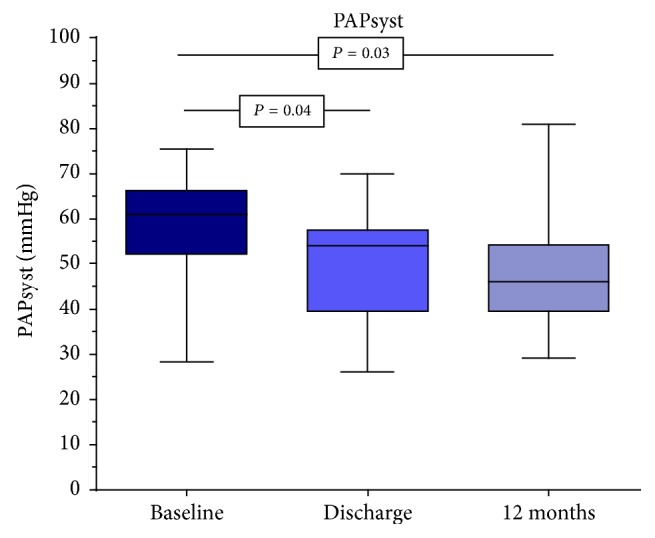
Decrease in systolic pulmonary artery pressure from 59 mmHg to 47 mmHg after twelve months.

**Figure 3:**
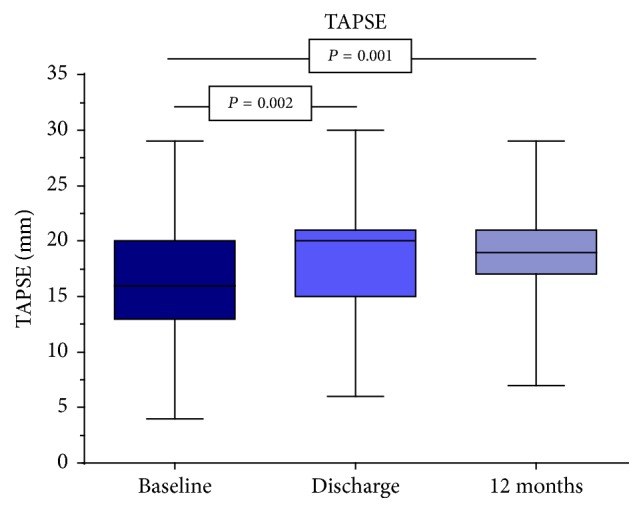
Increase of tricuspid annular plane systolic excursion (TAPSE) from 16.5 mm to 18.9 mm after 12 months.

The systolic and diastolic distance measurements of the TV annulus and the determination of the TR-Regurgitation area compared to the other time points remained unchanged within the observation period. Immediately after successful intervention, the vena contracta of the TR diminished from 0.88 cm to 0.82 cm, according to the RVOT. But a statistically significant value of 0.77 cm was reached after 12 months. In contrast, immediately after the intervention, there was a significant reduction in the maximum velocity of the TR from 4.17 m/s to 3.11 m/s, of the maximum gradient of the TR from 48.5 mmHg to 39.3 mmHg, and of the systolic pulmonary artery pressure from 58.6 mmHg to 50.0 mmHg. This decline continued in the following months (*V*_max_ TR to 3.09 m/s, peak TR to 38.6 mmHg, and PAPsyst to 47.4 mmHg), yet without becoming significant again compared to the values determined at discharge (Figure 2). Though, the significance in comparison to the results before implantation was continued. The TAPSE measurements revealed a similar picture. Immediately after the intervention, a significant increase from 16.5 mm to 18.1 mm was observed and to 18.9 mm after 12 months (Figure 3). However, statistically after one year, no difference existed between the measurements at the time of discharge and the representation of the patients after 12 months.

## 4. Discussion

Our results demonstrate that a significant reduction in systolic pulmonary artery pressure and the flow velocity of the tricuspid regurgitation follows immediately after successful MitraClip implantation. A Swiss research group observed a comparable immediate effect on the pulmonary artery pressure [22]. Both the pulmonary artery pressure and the flow reduced further during the 12-month follow-up observations. However, there was no longer any significant difference compared to the examination before dismissal. In addition, in our patient cohort, a significant increase of TAPSE occurred as a sign of improvement of the right ventricular contractility after successful MitraClip implantation. Giannini et al. could indicate a similar development in their group of patients in pulmonary artery pressure and TAPSE within a six-month observation period [23]. The pronounced reduction in pulmonary arterial pressure and the tricuspid regurgitation velocity and the increase in TAPSE, respectively, immediately after MitraClip implantation therefore most likely depend on smaller left ventricular or atrial regurgitation volumes after successful minimally invasive repair of the mitral valve and simultaneously reduced pressure load on the pulmonary circulation. Pulmonary vascular remodeling processes or adaptations requiring significantly more time appear to play no significant role in our patient group. These positive acute right ventricular changes are not concordant with the results of the EVEREST study [24]. No significant changes in either systolic or diastolic pulmonary artery pressure were observed immediately after MitraClip implantation there. This discrepancy can best be explained by a different composition of the patient cohort and study conditions. While in the EVEREST study primarily patients with a primary (degenerative) mitral regurgitation and very strict inclusion criteria regarding left ventricular function and left ventricular diameter and the valve morphology were enrolled [24–27], our cohort of patients consisted mainly of patients with secondary mitral regurgitation and a severely impaired left ventricular function with high NYHA classes. In addition, the hemodynamic measurements in the EVERST study were still collected during the general anesthesia necessary for the MitraClip implantation, so that here, in contrast to our patients who were examined after intervention in the waking state, there may be a possible reason for the different results. Patients with chronic reduced systolic LV ejection fraction who have abnormal TAPSE at baseline but reverse their RV dysfunction during follow-up by pharmacological optimization have better survival than patients with either worsened TAPSE or persistently abnormal TAPSE [28]. To what extent an improvement in TAPSE achieved by successful minimal invasive Mitral Valve Repair using MitraClip may lead to better long-term survival in our patient cohort as well is not known to date but is currently being analyzed. Preliminary data of our patients demonstrate similar results. In addition to the significant reductions or improvements experienced directly after the MitraClip intervention, comparably significant decreases in both the width of the vena contracta of the Tricuspid valve insufficiency and the diameter of the RVOT are brought about. A statistical significance of this reduction will only be reached after 12 months, so that the slower reduction is not immediately explicable with the decrease of pulmonary volume overload alone. In this case, it may have occurred during the postop observation time as a conversion/renaturation in the pulmonary circulation and a positive remodeling of the right ventricle, which needs more time to develop fully. These observations relate to the results of two working groups, which showed no significant changes in RV diameter in a follow-up period of six months [23, 29]. With respect to a reduction of the width of the vena contracta, hemodynamic relief of the right ventricle and the diameter of the RVOT after six months appears to be so insufficient as to be statistically significant.

However, in contrast to the above parameters, no significant decrease of the remaining right ventricular and atrial diameters could be documented in our patient cohort, even in the extended follow-up period of twelve months. The width of the tricuspid valve ring and the diameter of the inferior vena cava did not change in our group either. The reason for this is not clear. To what extent an even longer follow-up period of two or more years would result in significant changes remains speculative and should be investigated by further multicenter long-term studies.

## 5. Conclusion

Mitral Valve Repair by MitraClip improves the pulmonary artery pressure and tricuspid regurgitation in preexisting secondary pulmonary hypertension after 12 months. At the same time, there is a decrease in the diameter of the right ventricular outflow tract without significant changes in other right ventricular and right atrial dimensions. The TAPSE as a sign of right ventricular function also improves significantly within the follow-up period.

## Figures and Tables

**Table 1:** Demographic and clinical characteristics of all patients at baseline.

Mean age (yrs)	72,5 ± 9
Male	66%
NYHA functional class II	4 (6%)
NYHA functional class III	43 (61%)
NYHA functional class IV	23 (33%)
Kidney disease	66%
Atrial fibrillation	64%
Coronary artery disease	36%
Diabetes mellitus	27%
6-minute walk distance (m)	213 ± 54
Log EuroScore	30 ± 12
STS score	10 ± 4
Mitral regurgitation etiology	
Degenerative	20 (29%)
Functional	50 (71%)

**Table 2:** All measured echocardiographic parameters are shown as median and interquartile range in brackets.

Variable	Baseline	Discharge	*P* value	12 months	*P* value
RVD1 (cm)	4.27 (3.84; 4.59)	4.47 (3.91; 4.63)	0.16	4.48 (3.94; 4.59)	0.20
RVD2 (cm)	2.40 (2.07; 2.78)	2.58 (2.21; 2.99)	0.14	2.45 (2.20; 2.71)	0.38
RVD3 (cm)	6.03 (5.38; 6.70)	6.08 (5.47; 6.66)	0.89	5.97 (5.30; 6.55)	0.36
RV area (cm^2^)	19.8 (15.8; 23.4)	22.7 (17.0; 23.4)	0.39	19.7 (15.1; 22.1)	0.58
RAD (cm)	4.44 (3.88; 4.90)	4.45 (3.91; 4.80)	0.90	4.45 (4.00; 4.71)	0.71
RA area (cm^2^)	25.0 (21.2; 28.3)	25.6 (20.7; 28.8)	0.35	24.9 (20.9; 27.8)	0.37
IVC diameter (cm)	1.84 (1.55; 2.12)	1.86 (1.39; 2.21)	0.68	1.99 (1.74; 2.30)	0.16
RVOT (cm)	3.52 (3.09; 3.90)	3.44 (2.95; 3.85)	0.57	3.39 (3.13; 3.60)	0.01
TADes (cm)	3.13 (2.81; 3.38)	3.11 (2.85; 3.41)	0.75	3.21 (2.99; 3.50)	0.31
TADed (cm)	3.95 (3.59; 4.30)	4.04 (3.78; 4.34)	0.15	4.03 (3.60; 4.30)	0.70
Vena contracta (cm)	0.88 (0.65; 1.02)	0.82 (0.59; 0.89)	0.06	0.77 (0.61; 0.93)	0.01
Vmax TR (m/s)	4.17 (3.51; 4.73)	3.11 (2.78; 3.42)	0.001	3.09 (2.77; 3.27)	0.001
Peak TR (mmHg)	48.5 (41.2; 56.0)	39.3 (30.8; 46.4)	0.001	38.6 (30.7; 42.5)	0.001
PAPsyst (mmHg)	58.6 (52.1; 66.3)	50.0 (39.5; 57.5)	0.04	47.4 (39.5; 54.3)	0.03
TR area (cm^2^)	9.2 (4.9; 11.4)	9.0 (4.6; 11.3)	0.54	8.5 (5.0; 10.3)	0.69
TAPSE (mm)	16.5 (13.0; 20.1)	18.1 (15.0; 21.0)	0.002	18.9 (17.0; 21.3)	0.001

## Abbreviations

RV: Right ventricle
LV: Left ventricle
RA: Right atrium
TV: Tricuspid valve
IVC: Inferior vena cava
TR: Tricuspid regurgitation
PAPsyst: Systolic pulmonary artery pressure
TAPSE: Tricuspid annular plane systolic excursion
RVOT: Right ventricular outflow tract
RD1: Basal RV diameter
RD2: Mid RV diameter
RD3: RV base apex length
RAD: Right atrium diameter
*V*_max_ TR: Maximum velocity tricuspid regurgitation
Peak TR: Peak pressure tricuspid regurgitation
TADes: End-systolic tricuspid annular diameter
TADed: End-diastolic tricuspid annular diameter.
